# Sex-mediated effects of transglutaminase 2 inhibition on endothelial function in human resistance arteries from diabetic and non-diabetic patients

**DOI:** 10.1042/CS20242001

**Published:** 2025-01-15

**Authors:** Khatera Saii, Judit Prat-Duran, Ulf Simonsen, Anders Riegels Knudsen, Jonas Amstrup Funder, Niels Henrik Buus, Estéfano Pinilla

**Affiliations:** 1 Personalised Medicine, Department of Biomedicine, Aarhus University, Denmark; 2 Department of Abdominal Surgery, Aarhus University Hospital, Denmark; 3 Department of Clinical Medicine, Aarhus University; 4 Department of Renal Medicine, Aarhus University Hospital, Aarhus, Denmark

**Keywords:** Endothelium, Transglutaminase, Sex differences, Diabetic complications, Vascular function

## Abstract

Transglutaminase 2 (TG2) is an enzyme with multiple conformations. In its open conformation, TG2 exhibits transamidase activity linked to fibrosis, arterial remodeling, and endothelial dysfunction, a process enhanced by high glucose in endothelial cells. However, the closed conformation of TG2 contributes to transmembrane signaling and nitric oxide (NO)-dependent vasorelaxation. LDN 27219, a reversible allosteric inhibitor, stabilizes TG2 in its closed conformation. We examined whether pharmacological modulation of TG2 into its closed conformation induces vasorelaxation and enhances endothelium-dependent and independent relaxation in resistance arteries from age-matched diabetic (*n* = 14) and non-diabetic patients (*n* = 14) (age 71 (Standard Error of the Mean: ± 2)). Subcutaneous arteries (diameter 133–1013 µm) were isolated from abdominal fat biopsies. TG2 mRNA expression and transamidase activity were assessed via RT-qPCR and 5-biotin(amido)pentylamine (5-BP) incorporation, while vascular reactivity was measured using wire myography. TG2 mRNA was highly expressed without significant differences between the groups and LDN 27219 induced concentration-dependent vasorelaxation in arteries from both groups. Sex-specific analysis revealed that potentiation of acetylcholine-induced vasorelaxation by LDN 27219 was driven by increased TG2 expression in non-diabetic females, whereas no effect was observed in arteries from non-diabetic males. Among diabetic patients, LDN 27219 increased maximal acetylcholine-induced vasorelaxation in males only. LDN 27219 did not affect endothelium-independent relaxation to sodium nitroprusside in either group. In conclusion, TG2 is expressed in human resistance arteries, and LDN 27219 induced vasorelaxation, selectively enhancing ACh relaxation in non-diabetic females, likely owing to increased TG2 expression. This finding underscores the importance of sex differences in TG2 modulation of vasorelaxation.

## Introduction

Cardiovascular risk factors, including diabetes, hypertension, dyslipidemia, and smoking, accelerate the aging process and contribute to age-associated alterations such as endothelial dysfunction and stiffness in the vasculature [1]. These alterations can eventually lead to atherosclerotic vascular complications, such as coronary and peripheral vascular disease, as well as nephropathy [2].

Both type 1 and 2 diabetes are associated with microvascular complications, and the severity of the vascular dysfunction is dependent on factors such as disease duration and lack of glycemic control [2]. Moreover, insulin resistance and chronic exposure to hyperglycemia are independent risk factors for endothelial cell apoptosis, reduced nitric oxide (NO) bioavailability, and decreased sensitivity to NO at the vascular smooth muscle cell level [3]. NO bioavailability also decreases with aging, which reduces vascular relaxation in aging and diabetes [4].

The transglutaminase family of enzymes (TG1-7 and factor XIII) have transamidase activity, catalyzing the cross-linking of glutamine and lysine protein residues to form N epsilon (gamma-glutamyl) lysyl isopeptide bonds. Transglutaminase 2 (TG2) is the most widely expressed member of the family [5], and it is highly expressed in vascular endothelial and smooth muscle cells, cardiomyocytes, and macrophages in the cardiovascular system [6]. These expression data come mainly from animal models, and although TG2 has been found in primary endothelial cells and smooth muscle cells from humans, only a few studies have studied the expression of transglutaminases in the cardiovascular system in the context of disease [7,8]. Particularly, data are lacking in the context of diabetes [9].

TG2 exists in different conformations and locations (both extracellularly and intracellularly). Its various functions depend on its location, conformation, and microenvironment, including redox state, availability of Ca^2+^, and nucleotides [10]. In the presence of Ca^2+^, TG2 acquires an open conformation that is responsible for its transamidase activity, which has been involved in the development of fibrosis, arterial remodeling, endothelial dysfunction, and hypertension [11–14]. Additionally, high extracellular glucose concentrations have been linked with increased transamidase activity of TG2 in vascular endothelial cells, inducing cell death and endothelial leakage [13,15,16].

The closed conformation of TG2 has GTP-binding activity, thus acting as a G-protein in transmembrane signaling [17]. The closed TG2 conformation is involved in the opening of potassium channels in the smooth muscle cells, which increases the sensitivity to NO, resulting in vasorelaxation. Previous studies suggest that arteries from aging animals have increased amounts of TG2 in their open conformation, most likely due to reduced NO bioavailability [4,18], and that allosteric modulation of TG2 to its closed conformation could restore age-related changes in endothelial function [14]. LDN 27219 is a potent, slow-binding, reversible inhibitor of TG2, with an IC_50_ value of 0.6 μM [19]. It docks to a GTP-binding-related site of TG2, promoting the closed state and improving the vascular smooth muscle responsiveness to NO through the opening of potassium channels [14].

The present study hypothesized that pharmacological modulation of TG2 to the closed conformation by LDN 27219 would decrease vascular tone and improve endothelium-dependent vasorelaxation in human arteries, and these effects would be more pronounced in arteries from diabetic patients.

## Methods and materials

### Study population

The study was approved by the Regional Ethics Committee, Central Denmark (Permission: 1–10-72-352-18), and conducted in accordance with the principles of the Helsinki Declaration II for medical research. All participants provided informed consent prior to participation. Subcutaneous resistance arteries were obtained from fat biopsies in the abdominal area of male and female patients undergoing planned surgery at the Department of Surgery, Aarhus University Hospital, between September 2021 and June 2022.

Initially, patients were selected based on age, targeting elderly participants (60–80 years) and young to middle-aged participants (25–45 years). However, only three younger patients were included due to the low number of younger individuals undergoing open abdominal surgery. In total, 71 patients agreed to participate, of whom 58 had viable arteries for the myograph experiments (see later). To achieve a balanced analysis, each type 2 diabetic patient with viable arteries was matched to a non-diabetic patient of similar age and health status. This resulted in 14 diabetic and 14 non-diabetic patients being included in the primary analysis.

Patient files were reviewed to gather information on sex, age, indication for surgery, history of hypertension or heart disease, cardiovascular medications (e.g., calcium channel blockers, angiotensin-converting enzyme inhibitors, angiotensin receptor antagonists, beta-blockers), diabetes medications (e.g., metformin, insulin, dipeptidyl peptidase-1 inhibitors, glucagon-like peptide 1 receptor agonists), ongoing chemotherapy, and smoking status.

### Materials

The physiological saline solution (PSS, pH 7.4) had the following composition (mmol/L): 119 NaCl, 4.7 KCl, 1.18 KH₂PO₄, 1.17 MgSO₄, 1.5 CaCl₂, 24.0 NaHCO₃, 0.026 EDTA, and 5.5 glucose. The following drugs were used: acetylcholine (ACh), sodium nitroprusside (SNP), Nω-Nitro-L-Arginine (L-NOARG), phenylephrine, and U46619 (9α-epoxymethanoprostaglandin F2α) from Sigma (St. Louis, MO, USA). LDN 27219 was obtained from Tocris Bioscience (Bristol, UK). Unless otherwise stated, all substances were dissolved in distilled water and further diluted as required. The DMSO concentration in the bath was maintained below 0.1%, which did not affect vessel contractility.

### Tissue preparations

Fat biopsies were placed in ice-cold PSS immediately after surgery, and the arteries were dissected and cleaned within 1 hour. The dissected arteries were used immediately for myograph experiments or 5-biotin(amido)pentylamine (5-BP) incorporation and/or stored in RNAlater solution for reverse transcription-quantitative polymerase chain reaction (RT-qPCR) or snap-frozen for transamidase activity measurement. The number of arteries harvested from each biopsy varied considerably. Priority was given to using arteries for myograph experiments, transamidase activity measurements, and RT-qPCR.

### RT-qPCR

Depending on sample availability, additional arteries were dissected from a subset of patients and stored in RNAlater solution (Sigma-Aldrich). The samples were kept at −21°C until extraction and purification. Tissue preparation followed previously established procedures [17]. Briefly, total RNA was extracted and purified using the Nucleospin RNA Plus Kit (TaKara Bio) following the manufacturer’s instructions. Complementary DNA (cDNA) was synthesized with SuperScript IV Reverse Transcriptase (Life Technologies, Invitrogen) in a thermal cycler (VWR-XT96).

Prior to sample analysis, primers were optimized, and the amplified products were sequenced to ensure target specificity. In addition to TG1 and TG2, expression studies of transglutaminases 3–7 were performed using conventional PCR to determine whether other transglutaminases were expressed. Conventional PCR was conducted in the VWR-XT96 thermal cycler with a 40-cycle protocol. The reaction products were separated by agarose gel electrophoresis and visualized under UV light.

mRNA expression levels of transglutaminases 1, 2, and 7, as well as endothelial NO synthase (eNOS) and glyceraldehyde-3-phosphate dehydrogenase (GAPDH), were quantified by qPCR. One large section of the subcutaneous human artery from each patient was stored in RNAlater for this purpose.

qPCR was performed using the AriaMX Real-Time PCR system with a 50-cycle protocol. Cycle threshold (Ct) values for the target genes were normalized to Ct values for the housekeeping gene GAPDH, and data were quantified using Agilent Aria 2.0 software (Stratagene, Agilent Technologies). Results are expressed as a ratio to GAPDH. The primers and probes used for PCR and qPCR are detailed in Supplemental Table S3.

The positive controls for qPCR were human skin RNA (540031, AH Diagnostics) for TG3 and TG7, human prostate RNA (QS0624, AH Diagnostics) for TG4, human kidney RNA for TG5, and HK-2 cells (ATCC) for TG6.

### Transamidase activity assay

Transamidase activity was measured *ex vivo* using a dot blot assay. This method is based on the incorporation of the transglutaminase substrate 5-BP into structural proteins [4,14].

Two resistance artery segments were dissected from each patient. One artery was incubated in PSS containing 5-BP (0.3 mM) and 1.5 mM Ca²^+^, whereas the other was incubated in PSS with 1.5 mM Ca²^+^ but without 5-BP, serving as a negative control. Both were continuously bubbled with 5% CO₂, 75% N₂, and 20% O₂ for 4 hours at 37°C to maintain a pH of 7.4. Following incubation, unreacted 5-BP was removed by rinsing the arteries with PBS, after which they were snap-frozen for subsequent protein extraction.

Tissue homogenization and protein extraction were performed using radioimmunoprecipitation assay (RIPA) lysis buffer (0.5 mmol/L Tris/HCl pH 7.4, 10 mmol/L EDTA, 1.5 mmol/L NaCl, 2.5% deoxycholic acid, 10% NP-40) containing Halt Protease and Phosphatase Inhibitor Cocktail (ThermoFisher Scientific, Massachusetts, U.S.A.). Total protein concentration was quantified using a modified Lowry method, and 10 μg of total protein was loaded onto a nitrocellulose membrane using the Bio-Rad Dot Blot apparatus (Bio-Dot® Apparatus).

The membrane was blocked with 5% bovine serum albumin (BSA) for 2 hours and then incubated with horseradish peroxidase (HRP)-conjugated streptavidin (Amersham Bioscience; 1:10,000 dilution in 0.5% BSA) for 2 hours to detect 5-BP incorporation. The membrane was washed in Tris-buffered saline containing 0.1% Tween 20 detergent, removed from the dot blot apparatus, and developed using an ECL-Plus kit (General Electric Healthcare, Copenhagen, Denmark). Images were captured using the PXi 4 Touch image analysis system (Syngene), and blots were quantified with GeneTools 4 software (Syngene), normalized to total protein content.

### Isometric tension studies

Arteries were cut into segments approximately 2 mm in length and mounted in microvascular myographs (Danish Myotechnology, Aarhus, Denmark) using two 40 μm wires. The arteries were progressively stretched to their optimal diameter for isometric tension recordings, set at 0.9 times the estimated internal diameter at 100 mmHg of transmural pressure [20]. The myograph bath, containing PSS, was heated to 37°C and continuously bubbled with 5% CO₂, 75% N₂, and 20% O₂ to maintain a pH of 7.4. The viability of the vessels was tested at the start of each experiment by assessing their vasoconstrictor response to a high K^+^-concentration solution (KPSS). KPSS had the same composition as PSS, except NaCl was replaced equimolarly with KCl. In initial experiments, arteries were contracted three times with noradrenaline (NA, 5 μM) as a standard starting protocol. However, desensitization to NA was observed in some arteries, leading to a switch to KPSS as the standard starting procedure.

### Experimental protocol

To examine the direct vasodilatory effects of the TG2 inhibitor LDN 27219, arteries were contracted with the thromboxane analog U46619 (3 × 10^−8^ – 5 × 10^−8^ mol/L), and concentration-response curves (CRCs) for LDN 27219 (10^−8^ – 10^−4^ mol/L) or the corresponding vehicle (DMSO) were constructed from the same patient. All experiments included a vehicle control with an equivalent amount of solvent (DMSO). For this series of experiments, U46619 was chosen as the vasoconstrictor because arteries maintained a more stable contraction in the presence of the vehicle (DMSO) compared with phenylephrine (Phe) pre-constricted arteries.

To investigate the involvement of the endothelial pathway in potentiating endothelium-dependent vasorelaxation, arteries were contracted with Phe (3 × 10^−8^ – 10^−5^ mol/L), and CRCs for ACh (10^−9^ – 3 × 10^−6^ mol/L) were constructed. This was performed after 25 minutes of incubation with either vehicle (DMSO), LDN 27219 (3 × 10^−6^ M), or the NO synthase inhibitor L-nitroarginine (L-NOARG) (10^−4^ M) in the same artery. The concentration of Phe is expressed as a range because different doses were used to achieve the same relative contraction as KPSS. This approach was implemented to account for individual variations in sensitivity to Phe among patients.

To assess the involvement of LDN 27219 in endothelium-independent relaxation, the same artery from each patient was incubated with either vehicle (DMSO) or LDN 27219 (3 × 10^−6^ M) for 25 minutes, followed by CRCs for the NO donor SNP (10^−9^ – 3 × 10^−6^ mol/L).

### Statistical analysis

Power calculations based on the effect size found in previous experiments using human arteries [14], power of 95%, and an alpha of 0.05, determined a desired sample size of 13 patients per group. The experiments and following analysis were done blind to the patient’s history, and the diabetes status of the patients was checked in the patient record only after the data analysis was completed. The data are expressed as means ± SEM, where n is the number of patients in each group. Concentration-response curves were analyzed by two-way analysis of variance (ANOVA) followed by Bonferroni post-test for multiple comparisons. *P*-value < 0.05 was considered significant. All analyses were performed using GraphPad Prism Software (version 7.02).

## Results

A summary of the age-matched patient characteristics is presented in Table 1 for diabetic and non-diabetic patients. Individual characteristics for each age-matched patient are detailed in Supplemental Table S1, whereas the characteristics of the remaining patients are provided in Supplemental Table S2.

**Table 1:** List of patient characteristics of matched diabetic and non-diabetic patients.

	Diabetic patients (*n* = 14)	Non-diabetic patients (*n* = 14)
Sex	Female: 7 Male: 7	Female: 6 Male: 8
Age (years)	71 (SEM: ± 2)	71 (SEM: ± 2)
Indication for surgery	Pancreatic cancer (*n* = 9), splenomegaly (*n* = 1), colon cancer (*n* = 2), liver cancer (*n* = 1), anal cancer (*n* = 1)	Pancreatic cancer (*n* = 4), hepatocellular carcinoma (*n* = 2), cholangiocarcinom (*n* = 2), duodenal cancer (*n* = 1), retroperitoneal lipoma (*n* = 1), gall-bladder cancer (*n* = 1), rectum cancer (*n* = 1), peritoneal pseudomyxoma (*n* = 1), peritoneal cancer (*n* = 1)
Hypertension (blood pressure ≥ 140/90 mmHg)	57%	64%
Heart disease	21%	29%
Cardiovascular medication	64%	64%
Diabetes medication	93%	0%
Ongoing chemotherapy	21%	7%
Smoking status	Smoker: 21% Earlier smoker: 21%	Smoker: 14% Earlier smoker: 29%

### Expression of tranglutaminases and eNOS in arteries from diabetic and non-diabetic patients

**Figure 1:**
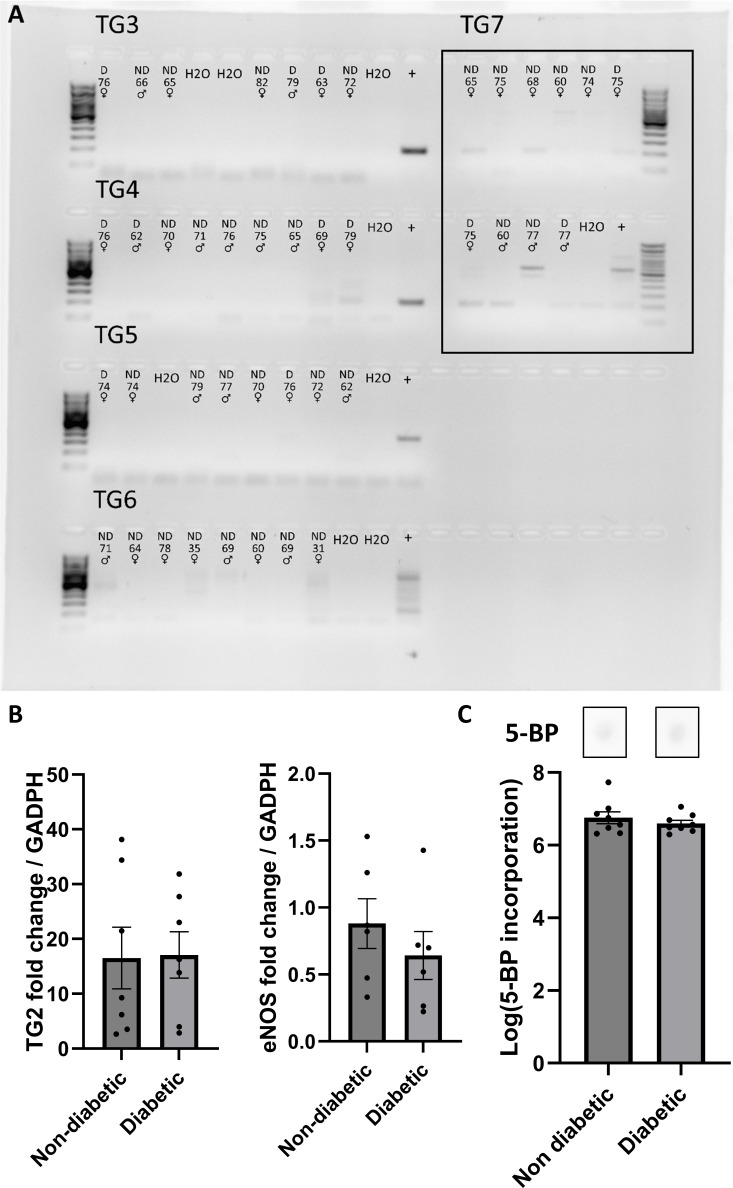
TG2 expression and activity and eNOS expression remain unchanged in diabetic patients. (**A**) Detection of transglutaminases 3–7 by qPCR. Each lane corresponds to samples from one patient; diabetic (**D**) or non-diabetic (ND) status is indicated together with the age and sex. Positive controls (+) were RNA of human Skin (AH diagnostics) for TG3 and TG7, human prostate total RNA (AH diagnostics) for TG4, human kidney from samples for TG5, HK-2 (ATCC) cells for TG6. The used primers can be found in Supplemental Table S3. (**B**) Quantification of mRNA expression relative to GAPDH by qPCR (**B**) of TG2 (left) and eNOS (right) in subcutaneous artery samples of age-matched diabetic and non-diabetic patients. (**C**) Transamidase activity quantified by 5-BP incorporation, representative blots (above), and average log (5-BP incorporation) (arbitrary units) (below) in subcutaneous artery samples of age-matched diabetic and non-diabetic patients. Data are mean + SEM.

The expression of transglutaminases in the resistance vasculature of humans has not been previously characterized. Although TG2 is the most abundantly expressed member of the family, other transglutaminases also present transamidase activity and may contribute to disease processes. To explore this, the presence of TG3 – TG7 mRNA was examined using conventional PCR in samples of subcutaneous arteries from diabetic and non-diabetic patients. Expression of TG7 was detected in arteries from five out of ten investigated patients (Figure 1A); therefore, we decided to quantify its expression using qPCR. None of the other isoenzymes were detected via conventional PCR.

The literature is unclear on whether differences in eNOS expression exist between diabetic and non-diabetic patients, but this could influence endothelium-dependent vasodilation. Therefore, eNOS expression was included in our qPCR experiments alongside TG1, TG2, and TG7, using GAPDH as the housekeeping gene. No differences in the expression of TG1, TG2, TG7, or eNOS mRNA were observed between diabetic and non-diabetic patients, either in the age-matched cohort (Figure 1B, Supplemental Figure S1A–C) or in the full cohort of studied patients (Supplemental Figure S1D–H).

### Transamidase activity in arteries from diabetic and non-diabetic patients

Transamidase activity was quantified by measuring 5-BP incorporation over 4 hours in arterial segments from diabetic and non-diabetic patients. No significant difference in transamidase activity was observed between diabetic and non-diabetic age-matched patients (Figure 1C).

### Direct vasorelaxant effects of LDN 27219

Human subcutaneous arteries (internal diameters ranging from 133 to 1013 μm, *n* = 104 arteries from 28 patients) with an average diameter of 551 μm±21, SEM were dissected from the biopsies. There was no significant difference in the average diameter between the arteries from diabetic (489 ± 25 μm, SEM) and non-diabetic (554 ± 25 μm, SEM) patients.

**Figure 2:**
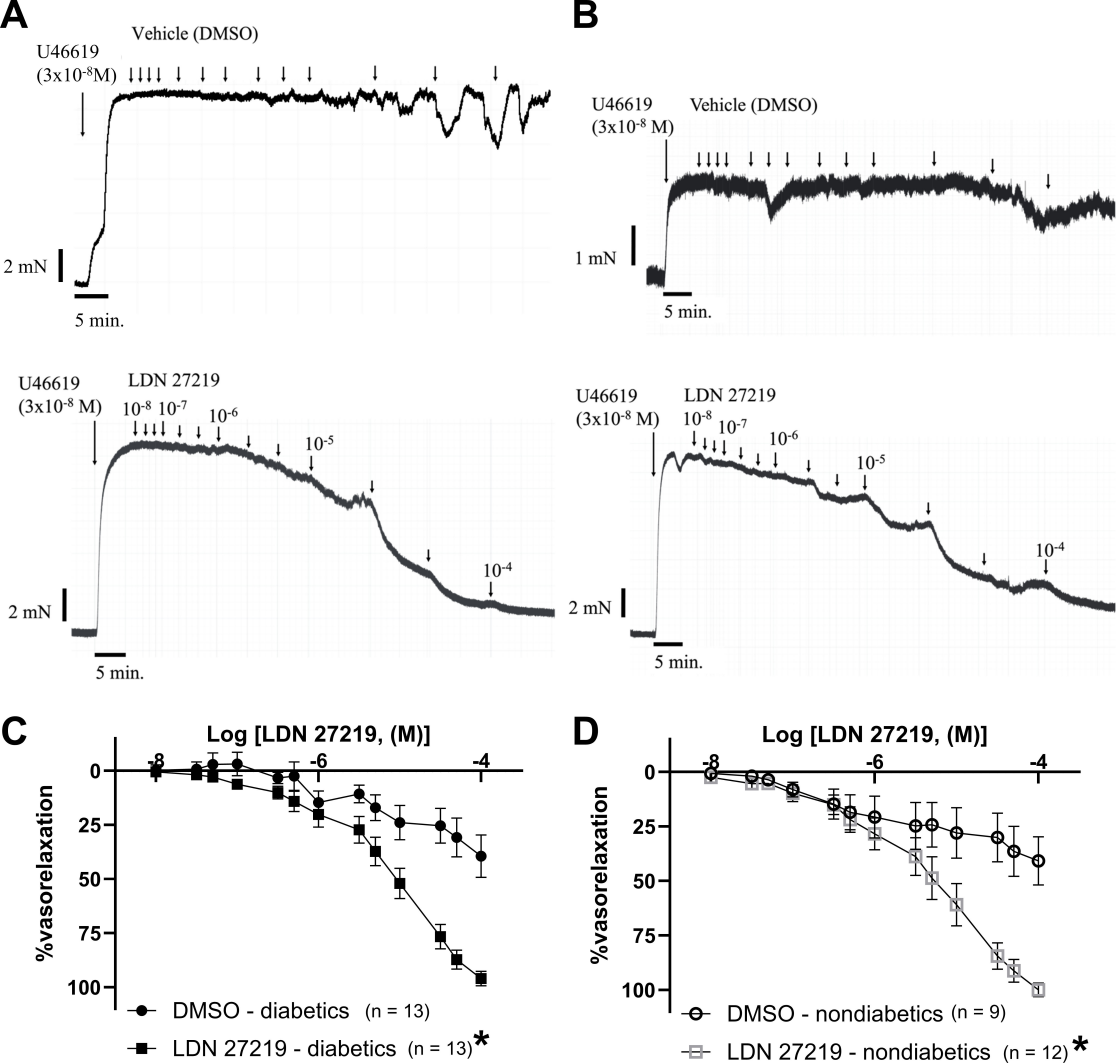
An allosteric modulator of TG2, LDN 27219 induces concentration-dependent relaxation of human subcutaneous resistance arteries. (**A–B**) Original traces in a diabetic patient (**A**) and a non-diabetic patient (**B**): showing contraction to the thromboxane analog U46619 (3 × 10^−8^ M), followed by a concentration-response curve for DMSO (vehicle) and below this for LDN 27219 (10^−8^ – 10^−4^ M). (**C-D**) Average concentration-response curves for LDN 27219 in arteries from diabetic (**C**) and non-diabetic patients (**D**). Data are mean + SEM. Differences were evaluated by two-way ANOVA *P < 0.05 versus DMSO vehicle.

LDN 27219 induced concentration-dependent relaxations in U46619-pre-constricted arteries from both diabetic and non-diabetic age-matched patients (Figure 2). The pre-constriction levels, EC_50_, and maximal relaxation values can be found in Supplemental Table S4. At the highest concentrations, LDN 27219 induced near-complete vasorelaxation regardless of diabetic status, with no significant difference observed in the maximal relaxation between the two patient groups.

### Effects of LDN 27219 on endothelium-dependent vasorelaxation

**Figure 3:**
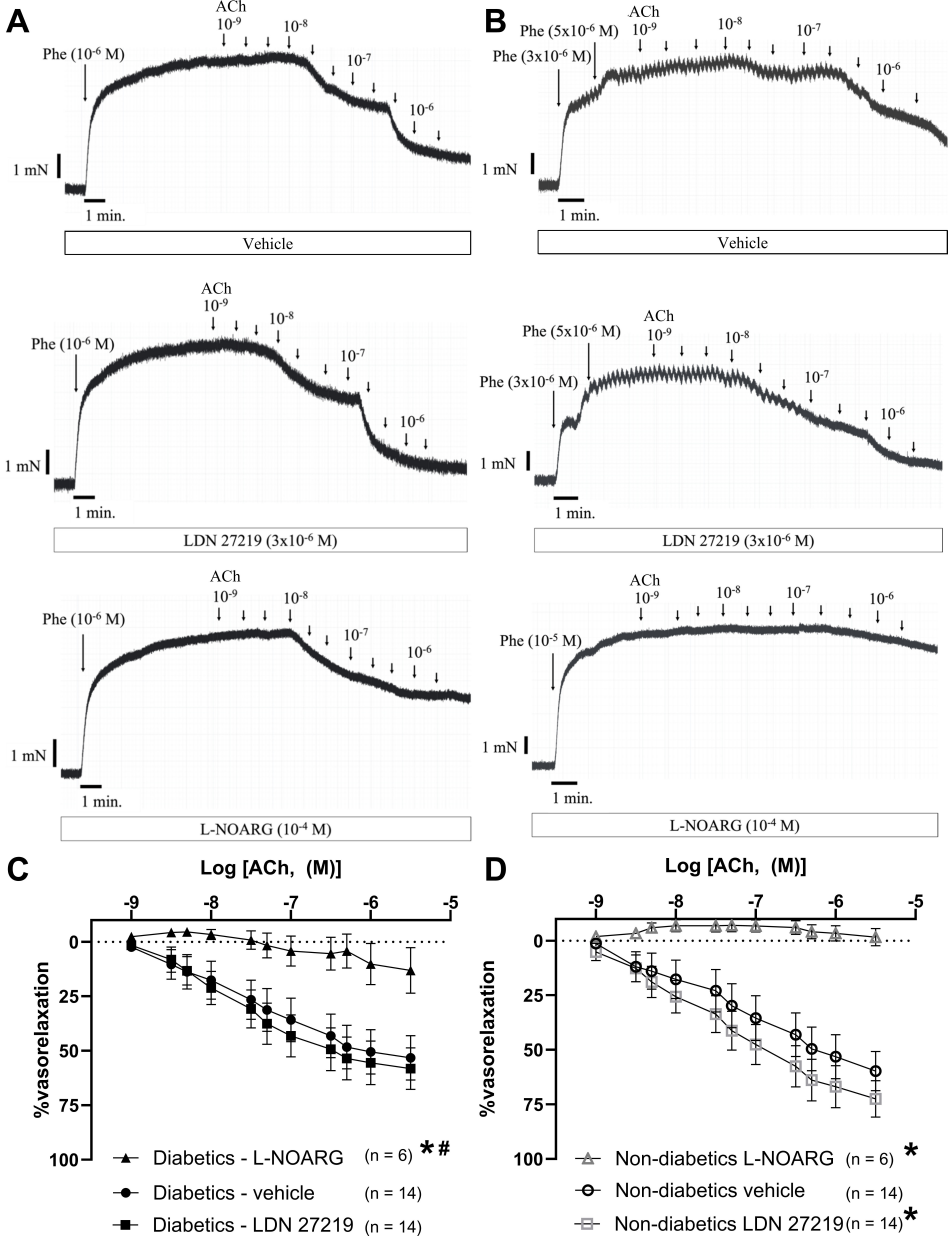
LDN 27219 potentiates endothelium-dependent relaxation in subcutaneous resistance arteries from non-diabetic patients. (**A–B**) Original traces showing arteries from diabetic (**A**) and non-diabetic (**B**) patients, which were incubated for 25 minutes with either vehicle (DMSO), LDN 27219 (3 × 10^−6^ M), or vehicle + L-nitroarginine (L-NOARG) (10^-4^ M). The arteries were contracted with Phe (5 × 10^−6^ – 10^−5^ M), followed by a concentration-response curve for ACh (10^−9^ – 3 × 10^−6^ M). (**C-D**) Average concentration-response curves for ACh in the diabetic (**C**) and the non-diabetic group (**D**). The non-diabetic group (**D**) had a significantly improved relaxation after incubation with LDN 27219 compared with the control curve (vehicle). Data are mean ± SEM. Differences were evaluated by two-way ANOVA *P < 0.05 versus vehicle; # *P* < 0.05 versus non-diabetic L-NOARG.

Arteries from diabetic and non-diabetic age-matched patients had similar endothelium-dependent vasorelaxation as demonstrated by comparable relaxations induced by ACh in Phe pre-constricted arteries. Incubation with LDN 27219 (3 × 10^−6^ M) significantly enhanced this response in the non-diabetic group (*P*<0.05 using two-way ANOVA) (Figure 3), particularly by increasing the maximal relaxation, while the EC₅₀ remained unaffected (Supplemental Table S4). In contrast, ACh-induced vasorelaxation remained unchanged in the diabetic group. Incubation with LDN 27219 (3 × 10^−6^ M) did not affect the pre-constriction of the artery (Supplemental Table S4).

To assess the contribution of NO to ACh-induced relaxation in each group, arteries were incubated with L-NOARG. This treatment significantly reduced ACh-induced vasorelaxation in both groups. However, the persistent relaxation in arteries from diabetic patients was significantly greater than in those from non-diabetic patients (Figure 3).

To assess whether the effect of LDN 27219 on ACh-induced vasorelaxation was because of the increased sensitivity of arterial smooth muscle to NO, we evaluated its impact on relaxation to the NO donor SNP. Arteries from both diabetic and non-diabetic patients exhibited similar responses to SNP, achieving near-complete relaxation, and incubation with LDN 27219 for 25 minutes did not alter SNP-induced vasorelaxation in either group (Supplemental Figure S2), suggesting that the primary effect of LDN 27219 in non-diabetic patients is not mediated through a direct increase in NO sensitivity.

### Impact of sex on the vascular effects of LDN 27219

**Figure 4:**
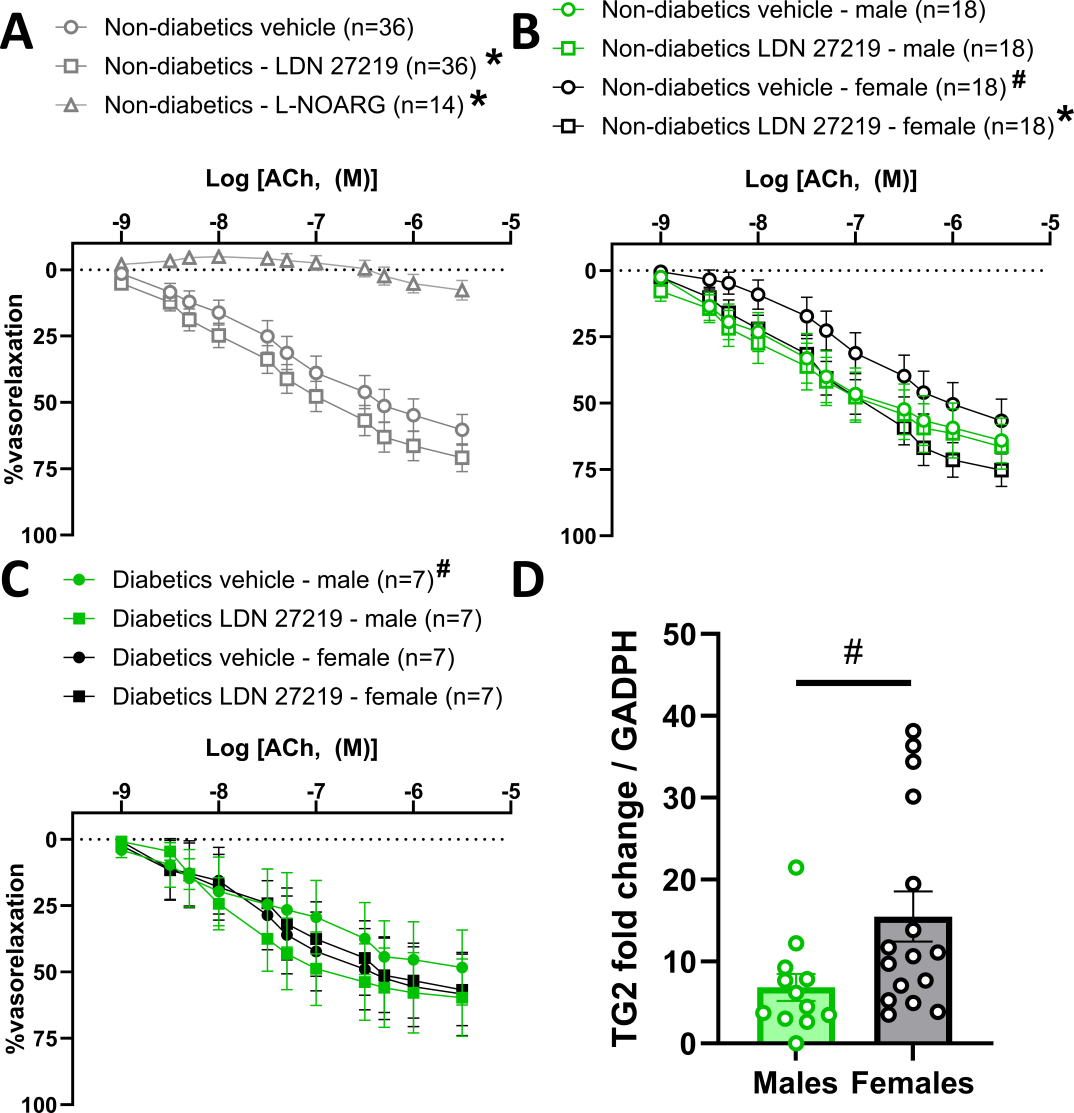
In all the non-diabetic patients included in the study, LDN 27219 potentiates endothelium-dependent vasorelaxation. However, this effect seems driven mainly by the arteries of female non-diabetic patients, which had a higher expression of (TG2 mRNA than those of male patients. (**A**) Average ACh concentration-response curves in arteries from all non-diabetic patients included in the study after incubation for 25 minutes with either vehicle (DMSO), LDN 27219 (3 × 10^−6^ M), or vehicle + L-nitroarginine (L-NOARG) (10^−4^ M). (**B–C**) Averages resulting from the same protocol in arteries from all non-diabetic (**B**) and diabetic (**C**) patients included in the study, separated by sex. (**D**) Quantification of TG2 mRNA expression relative to GAPDH by qPCR in subcutaneous artery samples of non-diabetic patients divided by sex. Data are mean ± SEM. Differences were evaluated by two-way ANOVA in the concentration-response curves and unpaired t-test in panel (**D**) *P < 0.05 versus vehicle; # *P*<0.05 versus non-diabetic male vehicle.

To confirm the effects of LDN 27219 on ACh-induced vasorelaxation in non-diabetic patients, we conducted a secondary analysis on the arteries of all 36 non-diabetic patients in the study. Note that the non-diabetic patients reported in Table 1 represent a subset of this larger group. Analysis of the entire non-diabetic patient data set confirmed that incubation with LDN 27219 increased ACh-induced vasorelaxation (Figure 4A). When sex was included as a variable, we observed that the effect of LDN 27219 on ACh-induced vasorelaxation in non-diabetic patients was primarily driven by female patients, as indicated by an increase in maximal relaxation, though logEC50 remained unchanged. In contrast, arteries from male patients were unaffected (Figure 4B and Supplemental Table S4). Additionally, arteries from non-diabetic female patients exhibited reduced vasorelaxation to ACh compared with those from non-diabetic male patients.

The results appeared reversed when sex was included in the analysis of arteries from diabetic patients. Arteries from male diabetic patients had reduced vasorelaxation compared with non-diabetic males, whereas this was not the case in arteries from female patients (Figure 4B–C). LDN 27219 did not affect ACh-induced vasorelaxation in arteries from diabetic females, but it increased maximal vasorelaxation to ACh in arteries from diabetic male patients (Supplemental Table S4). However, the concentration-response curve differences were insignificant when analyzed using two-way ANOVA (Figure 4C).

Sex-dependent effects were not detected on the direct vasorelaxant effect of LDN 27219, nor its effects on SNP-induced vasorelaxation. However, the inclusion of sex as a variable revealed that arteries from non-diabetic female patients had a reduced vasorelaxation response to SNP compared with males (Supplemental Figure S3).

Analysis of TG2 mRNA expression in the arteries of non-diabetic patients, separated by sex, showed significantly increased TG2 expression in female patients compared with male patients (Figure 4D), which could account for the observed sex-dependent effect of LDN 27219. No other significant sex-dependent differences were observed in the expression of other genes studied in diabetic or non-diabetic patients (Supplemental Figure S4A-E). Additionally, no significant sex differences were detected in transamidase activity, measured as 5-BP incorporation (Supplemental Figure S4D). However, it should be noted that the sample size for each group was limited after stratifying by sex.

## Discussion

Endothelial dysfunction is an independent predictor of vascular complications in diabetes [2], with TG2 activity playing a significant role, as demonstrated in various animal models of hyperglycemia [13,15,21]. However, the role of transglutaminases in human vascular tissue in relation to diabetes remains underexplored. In the present study, we identified TG2 as the most abundantly expressed transglutaminase mRNA species in human resistance arteries, followed by TG1 and TG7. We confirm that acute allosteric modulation of TG2 with LDN 27219 induces vasorelaxation in arteries from both diabetic and non-diabetic patients. However, the effects of TG2 modulation on endothelium-dependent vasorelaxation were observed only in non-diabetic patients. A secondary analysis incorporating sex as a variable revealed that this effect in non-diabetic patients was mainly driven by female patients, likely due to higher TG2 expression in their arteries. Additionally, a significant increase in maximal endothelium-dependent vasorelaxation following TG2 modulation was observed in male diabetic patients.

TG2 protein expression has been reported in human vascular tissue by immunohistochemistry in both endothelial and smooth muscle cells, mainly in the context of atherosclerosis [7,8], but the expression of other members of the transglutaminase family has not been studied, to the best of our knowledge. In the present study, we report the expression of TG1 mRNA for the first time, in addition to TG2. However, our results confirm that TG2 is the most abundantly expressed transglutaminase in the resistance vasculature of humans.

While the literature consistently reports decreased arterial NO production in diabetes, there is inconsistency regarding eNOS expression. Some studies indicate a reduction [22], while others find no change or even an increase in expression, but with reduced eNOS activation [23,24]. In our study, we did not observe differences in eNOS mRNA expression despite reduced endothelial function in male diabetic patients, which suggests reduced eNOS activation rather than altered expression, although we cannot discard a difference in expression at the protein level.

Previous studies have reported increased transamidase activity in endothelial cell cultures and murine models of hyperglycemia [15], whereas TG2 expression was unaltered in a human endothelial cell line [25], though we have recently found that there can be increased transamidase activity despite downregulation of TG2 [26]. In the present study, we did not detect differences in TG2 expression or total transamidase activity in subcutaneous arteries from diabetic and non-diabetic patients. This discrepancy may be due to previous experiments focusing on isolated endothelial cells, while our study assessed full arteries, where smooth muscle cells predominate. A lack of TG2 activation in smooth muscle layers could obscure endothelial-specific effects.

The magnitude of direct, concentration-dependent vasorelaxation caused by acute exposure to LDN 27219 was consistent with previous reports in arteries from rats, mice, and humans [14,21]. Our secondary analysis introducing sex as a variable revealed that, in non-diabetic patients, LDN 27219 potentiates ACh relaxation only in females, while in diabetic patients, LDN 27219 increased maximum vasorelaxation to ACh only in males, which presented reduced endothelium-dependent vasorelaxation. The effects in arteries from diabetic male patients are consistent with previous studies by our group describing increased potentiation of ACh vasorelaxation by LDN 27219 in resistance arteries of male aged and diabetic mice [21].

The presence of endothelial dysfunction in non-diabetic female patients, compared with non-diabetic males in our study, is unusual when considering the current literature. Based on the patients age (Supplemental Tables 1 and 2), it seems reasonable to assume that all non-diabetic female patients, except two, were post-menopausal. While the endothelial function of post-menopausal women has been shown to decrease markedly compared with pre-menopausal women, no differences have been reported between post-menopausal women and age-matched men in terms of endothelial dysfunction in previous studies [27]. However, it is important to note that all patients in this study, except three non-diabetic females and one diabetic female, were diagnosed with cancer and had received anti-cancer therapy at some point (Supplemental Tables 1 and 2). Such therapies are known to adversely affect cardiovascular health through mechanisms such as free radical production [28]. Emerging evidence from human and animal studies suggests that vascular endothelial cells in females may be particularly sensitive to this type of damage compared with male endothelial cells [29], which could explain our observations. Interestingly, our data suggest that, in our cohort of patients, diabetes may not exacerbate endothelial dysfunction in females, unlike in males.

The role of sex in TG2 regulation is understudied, with limited research addressing differential TG2 expression between males and females. In non-small cell lung cancer, cancerous tissue from female patients reportedly exhibited significant overexpression of TG2 compared with male patients. However, TG2 expression appeared to have greater prognostic significance in males, associated with higher recurrence rates and shorter disease-free survival, potentially indicating higher baseline expression in females [30]. In contrast, a study of clear cell renal cell carcinoma found that strong TG2 expression correlated with male sex [31], suggesting that sex-specific TG2 regulation may depend on the pathology and tissue involved. To our knowledge, we are the first to report sex-dependent regulation of TG2 in the vasculature. Our previous findings suggest a relationship between higher amounts of TG2 in its open conformation and reduced endothelium-dependent vasorelaxation [14]; therefore, the increased TG2 expression observed in resistance arteries from non-diabetic female patients may partially explain the reduced endothelium-dependent vasorelaxation in these arteries.

The main limitations of our study are the low number of diabetic patients we have in each group after stratifying for sex in the secondary analysis, which makes it difficult to reach definitive conclusions regarding mRNA expression and transamidase activity. It is also important to highlight that we only measured mRNA, which does not always correspond to the protein levels or activity in the tissue. This is illustrated by the fact that arteries from non-diabetic female patients exhibited higher TG2 mRNA expression compared with non-diabetic males (Figure 4D), but no difference in arterial TG2 enzyme activity was observed between the two groups (Supplemental Figure S4D). Additionally, we lack detailed information on diabetes duration in the patient records. Although some patients had a documented date of diagnosis, many had undiagnosed diabetes for an extended period, resulting in varying degrees of vascular damage. Moreover, we also lack information about HBA1c, which prevents us to evaluate if any patient had uncontrolled blood sugar levels. Another important limitation of the present study is that the arteries used for the transamidase activity and those used for the NO inhibition experiments were not age-matched due to the small sample size.

Regarding the potential off-target effects of LDN 27219 in the human arteries, we acknowledge that interactions beyond TG2 cannot be entirely ruled out. However, we believe that the primary mechanism underlying the observed vasoactive effects of LDN 27219 is its selective interaction with TG2, supported by several lines of evidence. First, hydrazine-based TG inhibitors, such as LDN 27219, exhibit high selectivity toward TG2 and TG3 [32] with minimal activity on other transglutaminase isozymes. In our study, we only observed minimal presence of TG1 and TG7, while TG2 expression is abundant and higher in non-diabetic females, where we observe the largest effect of LDN 27219. Lastly, in our previous studies on rat arteries and vascular smooth muscle cells, we found that the effects of LDN 27219, including BKCa channel activation, were fully inhibited by selective irreversible TG2 inhibitors, suggesting TG2 as the predominant target [14].

Finally, while our previous work demonstrates that the acute vascular effects of LDN 27219 in rats are primarily due to increased sensitivity to NO in vascular smooth muscle cells, this does not appear to be the case in human arteries, as evidenced by its lack of effect on SNP-induced vasodilatation. Future studies, such as investigating the impact of LDN 27219 on endothelium-denuded arteries, are necessary to uncover the exact mechanism of action of LDN 27219 in the human vasculature.

The primary findings of this study are that human resistance arteries have a high expression of TG2. LDN 27219 has an acute vasorelaxant effect in the arteries of both diabetic and non-diabetic patients. This direct effect was similar between the two groups. In arteries from non-diabetic patients, LDN 27219 potentiated endothelium-dependent vasorelaxation, while it failed to do so in diabetic patients. A sex-dependent component of the effect was identified in a secondary analysis, showing that the effect of LDN 27219 in arteries from non-diabetic patients was restricted to female patients only, likely due to their higher arterial expression of TG2. In contrast, LDN 27219 increased endothelium-dependent vasorelaxation in arteries from male diabetic patients. These findings underscore the importance of sex-specific differences in TG2 expression and function, particularly in non-diabetic individuals, and highlight their importance when exploring TG2-targeted therapies in vascular dysfunction.

**Clinical perspective**

- Transglutaminase 2 (TG2) activity contributes to endothelial dysfunction in the vasculature, a key independent predictor of vascular complications in diabetes, but the role of transglutaminases in human tissue remains underexplored.
- Our study shows that TG2 is the most abundantly expressed transglutaminase in human resistance arteries and that its modulation using an allosteric inhibitor induces vasorelaxation in the arteries of both diabetic and non-diabetic patients. Notably, this modulation significantly enhances endothelium-dependent vasorelaxation in female non-diabetic patients, who exhibit reduced vasorelaxation and higher TG2 expression compared with non-diabetic males. In diabetic patients, the effect is also significant, though with only a small increase in maximal endothelium-related vasorelaxation observed in male arteries.
- We report, for the first time, a sex-dependent effect of TG2 modulation on endothelium-dependent vasorelaxation. These findings underscore the importance of further exploring sex-specific differences in TG2 expression and function to better understand their implications for vascular health and to guide the development of TG2-targeted therapies.

## Supplementary material

Supplementary material

## Data Availability

The raw data that support the findings of the present study are available from the corresponding author upon request.

## Abbreviations

ACh: Acetylcholine
ACh: acetylcholine
5 BP: 5-biotin(amido)pentylamine
5-BP: 5-biotin(amido)pentylamine
BSA: Bovine Serum Albumin
BSA: bovine serum albumin
CRCs: concentration-response curves
CRCs: Concentration-response curves
KPSS: Physiological saline solution with high concentration of potassium
KPSS: K+-concentration
L-NOARG: Nω-Nitro-L-Arginine
L-NOARG: Nω-nitro-l-arginine
NA: Noradrenaline
NA: noradrenaline
NA: Noradrenaline
NO: Nitric Oxide
NO: nitric oxide
PSS: Physiological saline solution
PSS: physiological saline solution
Phe: phenylephrine
Phe: Phenylephrine
SNP: Sodium nitroprusside
SNP: sodium nitroprusside
TG2: Transglutaminase 2
TG2: Transglutaminase 2
