# Supplementary material for: Sex-mediated effects of transglutaminase 2 inhibition on endothelial function in human resistance arteries from diabetic and non-diabetic patients
Source: Clin Sci (Lond). 2025 Jan 15;139(1):1–14. doi: 10.1042/CS20242001 (PMC12204022; doi:10.1042/CS20242001)
Supplement: Supplementary material [file CS-139-01-CS20242001-s001.docx]

**SUPPLEMENTAL TABLES**

**Table S1**: *Characteristics of age-paired human donors of fat biopsies with contractile vessels, paired patients appear consecutively on the table.*

| **Patient group** | **Sex** | **Age (years)** | **Reason for surgery** | **BMI (kg/m^2^)** | **Hyper-tension** | **Heart-disease** | **CV medication** | **DM medication** | **Ongoing chemo-therapy** | **Smoking status** |
| --- | --- | --- | --- | --- | --- | --- | --- | --- | --- | --- |
| Diabetic | Female | 76 | Pancreatic cancer | 29.4 | Yes | Yes | Yes | No | n.a. | No |
| Non | Male | 77 | Hepatocellular carcinoma | n.a. | Yes | No | Yes | No | No | Earlier |
| diabetic |  |  |  |  |  |  |  |  |  |  |
| Diabetic | Male | 79 | Pancreatic cancer | 23.2 | No | No | Yes | Yes | Yes | Yes |
| Non | Male | 76 | Colon cancer | n.a. | Yes | No | No | No | No | No |
| diabetic |  |  |  |  |  |  |  |  |  |  |
| Diabetic | Female | 79 | Pancreatic cancer | 22.4 | No | No | Yes | Yes | No | No |
| Non | Female | 78 | Cholangio-carcinoma | n.a. | No | No | No | No | No | No |
| diabetic |  |  |  |  |  |  |  |  |  |  |
| Diabetic | Female | 69 | Pancreatic cancer | n.a. | Yes | No | No | Yes | No | Yes |
| Non | Male | 69 | Hepatocellular carcinoma | n.a. | No | No | Yes | No | Yes | Yes |
| diabetic |  |  |  |  |  |  |  |  |  |  |
| Diabetic | Male | 62 | Pancreatic cancer | 21.3 | Yes | No | Yes | No | No | No |
| Non | Male | 62 | Duodenal cancer | 25.1 | Yes | Yes | Yes | No | No | No |
| diabetic |  |  |  |  |  |  |  |  |  |  |
| Diabetic | Female | 63 | Pancreatic cancer | 29 | No | No | No | Yes | Yes | Earlier |
| Non | Female | 64 | Retro-peritoneal lipoma | n.a. | Yes | No | Yes | No | No | No |
| diabetic |  |  |  |  |  |  |  |  |  |  |
| Diabetic | Female | 76 | Spleno-megaly | 35.1 | Yes | No | Yes | Yes | No | No |
| Non | Male | 76 | Pancreatic cancer | n.a. | Yes | No | Yes | No | No | No |
| diabetic |  |  |  |  |  |  |  |  |  |  |
| Diabetic | Female | 75 | Pancreatic cancer | 23.6 | No | No | No | Yes | No | No |
| Non | Male | 75 | Pancreatic cancer | n.a. | Yes | No | Yes | No | No | No |
| diabetic |  |  |  |  |  |  |  |  |  |  |
| Diabetic | Male | 72 | Colon cancer | 27.2 | Yes | No | Yes | Yes | No | Yes |
| Non | Female | 72 | Gall-bladder cancer | n.a. | Yes | Yes | Yes | No | No | Earlier |
| diabetic |  |  |  |  |  |  |  |  |  |  |
| Diabetic | Male | 61 | Colon cancer | n.a. | Yes | No | Yes | Yes | No | No |
| Non | Female | 60 | Rectum cancer | n.a. | Yes | Yes | Yes | No | No | Yes |
| diabetic |  |  |  |  |  |  |  |  |  |  |
| Diabetic | Male | 67 | Pancreatic cancer | n.a. | Yes | Yes | Yes | Yes | No | No |
| Non | Female | 68 | Pseudo-myxoma | n.a. | No | No | No | No | No | No |
| diabetic |  |  |  |  |  |  |  |  |  |  |
| Diabetic | Male | 77 | Liver cancer | n.a. | Yes | Yes | Yes | Yes | No | Earlier |
| Non | Male | 77 | Pancreatic cancer | 24.7 | Yes | Yes | Yes | No | No | Earlier |
| diabetic |  |  |  |  |  |  |  |  |  |  |
| Diabetic | Male | 75 | Anal cancer | n.a. | No | No |  | Yes | Yes | No |
| Non | Female | 74 | Pancreatic cancer | n.a. | No | No | No | No | No | Earlier |
| diabetic |  |  |  |  |  |  |  |  |  |  |
| Diabetic | Female | 61 | Pancreatic cancer | 24.1 | No | No | No | Yes | No | Yes |
| Non | Male | 60 | Peritoneal cancer | n.a. | No | No | No | No | No | No |
| diabetic |  |  |  |  |  |  |  |  |  |  |

BMI: Body mass index; CV: Cardiovascular; DM: Diabetes Mellitus

**Table S2**: *Characteristics of the remaining human donors of fat biopsies with contractile vessels*

| **Patient group** | **Sex** | **Age (years)** | **Reason for surgery** | **BMI (kg/m^2^)** | **Hyper-tension** | **Heart-disease** | **CV medication** | **DM medication** | **Ongoing**  **chemo-therapy** | **Smoking status** |
| --- | --- | --- | --- | --- | --- | --- | --- | --- | --- | --- |
| Diabetic (T1D) | Female | 74 | Pancreatic cancer | 26.6 | No | Yes | No | Yes | No | No |
| Diabetic (T1D) | Female | 74 | Pancreatic cancer | n.a. | Yes | No | Yes | Yes | No | No |
| Non | Male | 64 | Intrahepatic cholangio-carcinoma | 28 | No | No | No | No | No | No |
| diabetic |  |  |  |  |  |  |  |  |  |  |
| Non | Female | 65 | Pancreatic cystic proces | 21.9 | No | No | No | No | No | No |
| diabetic |  |  |  |  |  |  |  |  |  |  |
| Non | Male | 66 | Gall-bladder cancer | 29 | No | No | No | No | No | No |
| diabetic |  |  |  |  |  |  |  |  |  |  |
| Non | Female | 82 | Hepatocellular carcinoma | n.a. | Yes | Yes | Yes | No | No | No |
| diabetic |  |  |  |  |  |  |  |  |  |  |
| Non | Male | 65 | Pancreatic cancer | 25 | Yes | No | Yes | No | No | No |
| diabetic |  |  |  |  |  |  |  |  |  |  |
| Non | Male | 76 | Pancretic cancer | 29.4 | Yes | Yes | Yes | No | No | Earlier |
| diabetic |  |  |  |  |  |  |  |  |  |  |
| Non | Male | 71 | Rectum cancer | 20,8 | No | No | No | No | No | No |
| diabetic |  |  |  |  |  |  |  |  |  |  |
| Non | Female | 70 | Bile duct cancer | 35 | Yes | No | Yes | No | No | No |
| diabetic |  |  |  |  |  |  |  |  |  |  |
| Non | Male | 74 | Pancreatic cancer | 29,8 | No | Yes | Yes | No | No | No |
| diabetic |  |  |  |  |  |  |  |  |  |  |
| Non | Male | 79 | Colon cancer | 21,6 | No | No | No | No | No | Earlier |
| diabetic |  |  |  |  |  |  |  |  |  |  |
| Non | Male | 77 | Hepatocellular carcinoma | 30 | Yes | Yes | Yes | No | No | Earlier |
| diabetic |  |  |  |  |  |  |  |  |  |  |
| Non | Female | 70 | Hepatocellular carcinoma | n.a. | No | No | No | No | No | No |
| diabetic |  |  |  |  |  |  |  |  |  |  |
| Non | Male | 71 | Colon cancer | n.a. | No | No | No | No | No | No |
| diabetic |  |  |  |  |  |  |  |  |  |  |
| Non | Female | 35 | Gall-bladder dysplasia | n.a. | No | No | No | No | No | No |
| diabetic |  |  |  |  |  |  |  |  |  |  |
| Non | Male | 69 | Gall-bladder cancer | 28,7 | Yes | No | No | No | No | No |
| diabetic |  |  |  |  |  |  |  |  |  |  |
| Non | Male | 70 | Colon cancer | n.a. | Yes | Yes | Yes | No | No | No |
| diabetic |  |  |  |  |  |  |  |  |  |  |
| Non | Female | 60 | Leiomyo-sarcoma | n.a. | No | No | No | No | No | No |
| diabetic |  |  |  |  |  |  |  |  |  |  |
| Non | Female | 64 | Benign polyp | n.a. | No | No | No | No | No | No |
| diabetic |  |  |  |  |  |  |  |  |  |  |
| Non | Female | 31 | Hemangiom-endothelium | n.a. | No | No | No | No | No | No |
| diabetic |  |  |  |  |  |  |  |  |  |  |
| Non | Female | 65 | Pseudo-myxoma | n.a. | No | No | No | No | No | No |
| diabetic |  |  |  |  |  |  |  |  |  |  |
| Non | Female | 75 | Intra-abdominal tumor | n.a. | No | No | No | No | No | No |
| diabetic |  |  |  |  |  |  |  |  |  |  |
| Non | Female | 74 | NET-tumor | n.a. | Yes | No |  | No | No | No |
| diabetic |  |  |  |  |  |  |  |  |  |  |
| Non | Male | 64 | Colon cancer | n.a. | No | Yes | Yes | No | No | Earlier |
| diabetic |  |  |  |  |  |  |  |  |  |  |
| Non | Female | 67 | Splenomegaly | n.a. | No | No | No | No | No | No |
| diabetic |  |  |  |  |  |  |  |  |  |  |
| Non | Female | 45 | Colon cancer | n.a. | No | No | No | No | Yes | Earlier |
| diabetic |  |  |  |  |  |  |  |  |  |  |
| Non | Female | 64 | Hepatocellular carcinoma | 22,8 | No | No | No | No | No | Earlier |
| diabetic |  |  |  |  |  |  |  |  |  |  |
| Non | Female | 72 | Pancreatic cancer | n.a. | No | No | No | No | Yes | No |
| diabetic |  |  |  |  |  |  |  |  |  |  |
| Non | Male | 66 | Pancreatic cancer | n.a. | Yes | Yes | Yes | No | No | Earlier |
| diabetic |  |  |  |  |  |  |  |  |  |  |

BMI: Body mass index; CV: Cardiovascular; DM: Diabetes Mellitus; T1D: Type 1 Diabetes

**Table S3: Primers and probes for the RT-PCR and RT-qPCR**

Forward (5’-3’) Reverse (5’-3’) Probe (5’-3’)

| **Tgm1** | CACCAACTTCAACTCCGCCCA | CACCACCTGCCACCCATCAAA | ACAGACACATCCCTTACCATGG |
| --- | --- | --- | --- |
| **Tgm2** | ACCAGCAAGACTGCACCCTCT | AACTGCCCAAARTTCCAAGG | TCCAGCTTTGTGCTGGGCCACTTCAT |
| **Tgm3** | GCCAGTGCTGGGTCTTTGCT | AACCAGCCTTCATTCCAGAC |  |
| **Tgm4** | CAGAAGTGAATGGTGACAGG | TGAGAAGGAGGAAGGCATGA |  |
| **Tgm5** | GGTTGAGGACTGTGTGCTGAC | CCTTAATGTCCTTAAACTTGT |  |
| **Tgm6** | CGGAAAGAGAGGCAGGTGTA | AGCGATGCTGGGCTTGGTG |  |
| **Tgm7** | CCGGCCAAAGACTGTTCCCA | TAGTCCTCGCCCCAGTTCC |  |
| **Gadph** | CCCATGGCAAATTCCATGGCAC | TCCATGGTGGTGAAGACGCCA | TGTCATCAATGGAAATCCCATCACCA |
| **eNOS** | TGTGACCCTCACCGCTACAAC | CCCCTGGCCTTCTGCTCATTCT | TCCATGCAGACAGCCACATCCTC |

**Table S4: Level of contraction induced by the vasoconstrictors and Emax and pD2 values of different vasodilators.**

| **Figure** | **Conditions** | **n** | **Vasoconstrictor** | **Contraction level (%KPSS)** | **Vasodilator** | **E_max_ (Relaxation %)** | **logEC50** |
| --- | --- | --- | --- | --- | --- | --- | --- |
| Figure 2C | Diabetic | 13 | U46619 3x10^-9^ M | 95,3 ± 28,8 | DMSO | 39,5 ± 9,2 | ND |
|  | Diabetic | 13 |  | 98,8 ± 32,4 | LDN 27219 | 96 ± 3,3 | -5 |
|  |  |  |  |  |  |  | (-5,2 to -4,9) |
| Figure 2D | Non-diabetic | 9 | U46619 3x10^-9^ M | 102,8 ± 19,5 | DMSO | 40,7 ± 10,9 | ND |
|  | Non-diabetic | 12 |  | 115,5 ± 17,9 | LDN 27219 | 99,9 ± 3 | -5,2 |
|  |  |  |  |  |  |  | (-5,4 to -5,1) |
| Figure 3C | Diabetic vehicle | 14 | Phe 5x10^-6^-10^-5^M | 89,8 ± 13,8 | ACh 10^-9^ – 3x10^-6^ M | 53,2 ± 10,2 | -7,4 |
|  |  |  |  |  |  |  | (-8,2 to -6,7) |
|  | Diabetic LDN 27219 | 14 |  | 85,1 ± 19,2 |  | 58,1 ± 9,4 | -7,6 |
|  |  |  |  |  |  |  | (-8,2 to -7) |
|  | Diabetic L-NOARG | 6 |  | 99,5 ± 25,3 |  | 13 ± 10,4* | ND |
| Figure 3D | Non-diabetic vehicle | 14 | Phe 5x10^-6^-10^-5^M | 88,2 ± 14,8 | ACh 10^-9^ – 3x10^-6^ M | 59,8 ± 8,9 | -7,1 |
|  |  |  |  |  |  |  | (-7,8 to -6,3) |
|  | Non-diabetic | 14 |  | 80,4 ± 17,4 |  | 72,5 ± 8,3* | -7,3 |
|  | LDN 27219 |  |  |  |  |  | (-7,8 to -6,8) |
|  | Non-diabetic | 6 |  | 96,6 ± 25,7 |  | -1,7 ± 3,9* | ND |
|  | L-NOARG |  |  |  |  |  |  |
| Figure 4A | Non-diabetic vehicle | 36 | Phe 5x10^-6^-10^-5^M | 94,7 ± 4,7 | ACh 10^-9^ – 3x10^-6^ M | 60,3 ± 5,8 | -7,3 |
|  |  |  |  |  |  |  | (-7,6 to -6,9) |
|  | Non-diabetic | 36 |  | 89,8 ± 5,2 |  | 70,8 ± 5,1* | -7,4 |
|  | LDN 27219 |  |  |  |  |  | (-7,7 to -7,1) |
|  | Non-diabetic | 14 |  | 113,2 ± 8,6 |  | 7,6 ± 3,6* | ND |
|  | L-NOARG |  |  |  |  |  |  |
| Figure 4B | M- Non-diabetic | 18 | Phe 5x10^-6^-10^-5^M | 102,1 ± 7,2 | ACh 10^-9^ – 3x10^-6^ M | 64 ± 8,4 | -7,5 |
|  | vehicle |  |  |  |  |  | (-8,2 to -7) |
|  | M- Non-diabetic | 18 |  | 93 ± 8,6* |  | 66,4 ± 8,3 | -7,5 |
|  | LDN 27219 |  |  |  |  |  | (-8,1 to -6,9) |
|  | F- Non-diabetic | 18 |  | 87,4 ± 5,7 |  | 56,6 ± 8,1 | -7,1 |
|  | Vehicle |  |  |  |  |  | (-7,5 to -6,6) |
|  | F- Non-diabetic | 18 |  | 86,6 ± 6 |  | 75,2 ± 6,1* | -7,3 |
|  | LDN 27219 |  |  |  |  |  | (-7,6 to -7) |
| Figure 4C | M- Diabetic | 7 | Phe 5x10^-6^-10^-5^M | 100,2 ± 10,4 | ACh 10^-9^ – 3x10^-6^ M | 48,2 ± 13,9 | -7,3 |
|  | vehicle |  |  |  |  |  | (-8,8 to -5,9) |
|  | M- Diabetic | 7 |  | 98,4 ± 14,4 |  | 59,6 ± 14,5* | -7,8 |
|  | LDN 27219 |  |  |  |  |  | (-8,5 to -7,1) |
|  | F- Diabetic | 7 |  | 76,7 ± 6,9 |  | 54,4 ± 13 | -7,4 |
|  | vehicle |  |  |  |  |  | (-8,2 to -6,6) |
|  | F- Diabetic | 7 |  | 69,23 ± 11 |  | 55,1 ± 11,8 | -7,3 |
|  | LDN 27219 |  |  |  |  |  | (-8,2 to -6,5) |

Values are means ± SEM or means 95% CI interval, and are calculated from the data in figures indicated in the left column. n: number of patients. *, P < 0.05 compared to control/vehicle conditions using Wilcoxon matched-pairs signed rank test. M: Male. F: Female. ND: Not determined because Emax was less than 50%

**SUPPLEMENTAL FIGURES**


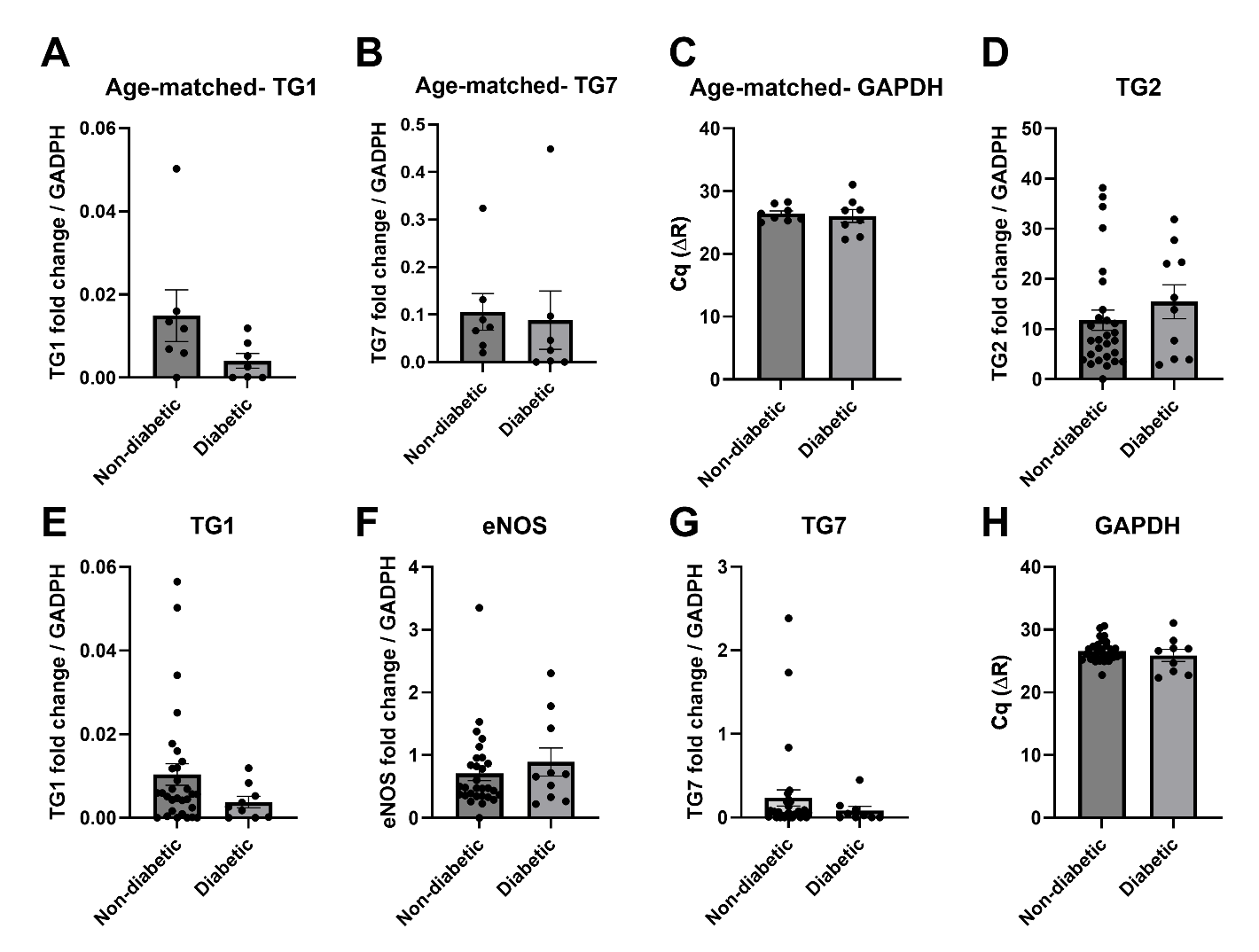


**Supplemental Figure S1**: Quantification of mRNA expression by RT-qPCR of TG1 (A), TG7 (B), GAPDH (C) in subcutaneous artery samples of age-matched diabetic and non-diabetic patients, and expression of TG2 (D), TG1 (E), eNOS (F), TG7 (G) and GADPH (H) in subcutaneous arteries of all studied patients. Data are averages ± SEM.


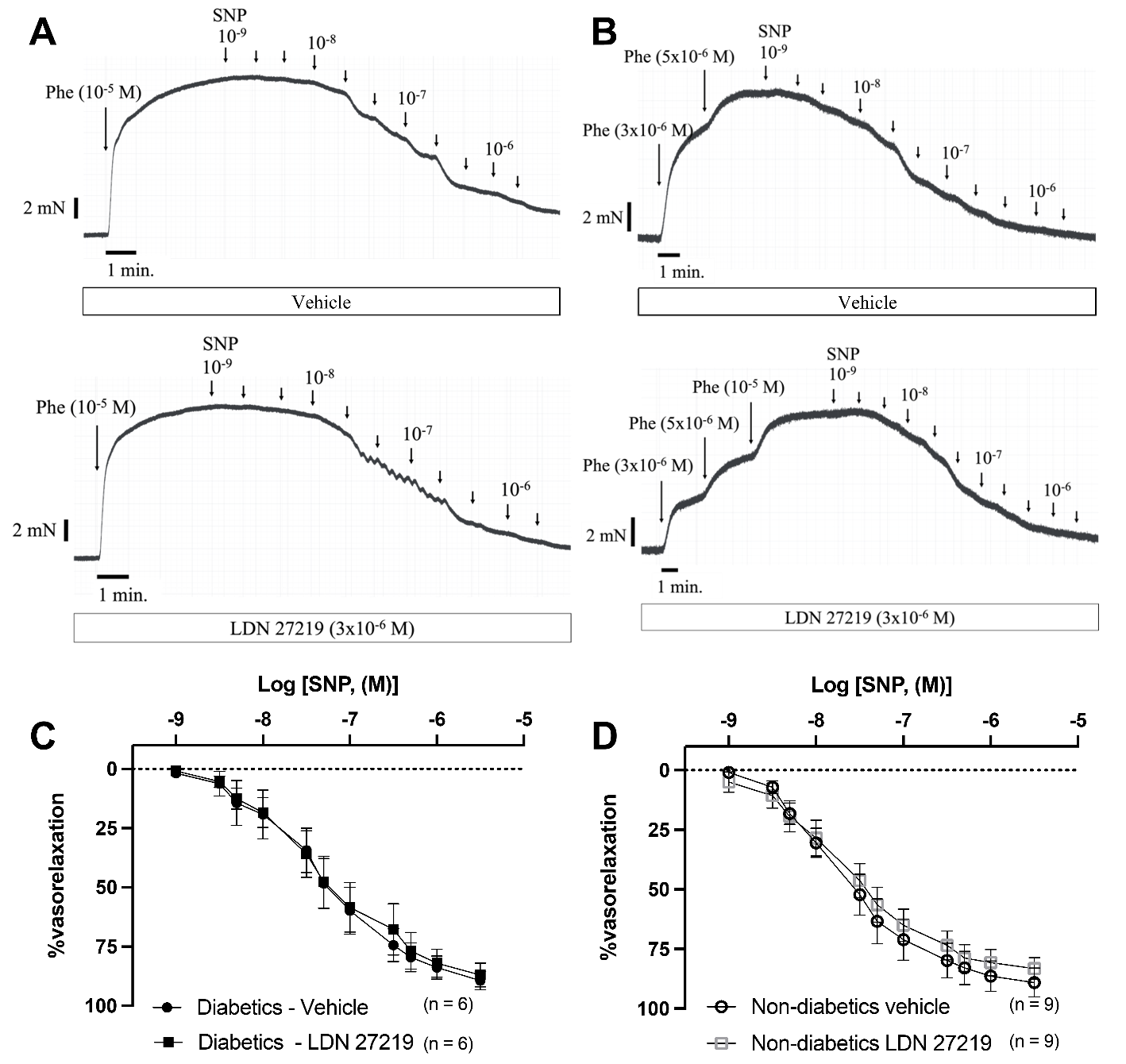


**Supplemental Figure S2**: No effect of LDN 27219 on relaxations induced by sodium nitroprusside (SNP) in human subcutaneous resistance arteries. **A-B.** Original traces showing arteries from a diabetic (A) and nondiabetic (B) patient, which were incubated for 25 minutes with either vehicle (DMSO) or LDN 27219 (3x10^-6^ M). The arteries were contracted with Phe (5x10^-6^–10^-5^ M), followed by a concentration-response curve for SNP (10^-9^ – 3x10^-6^ M). **C-D.** Average vasorelaxations to SNP in arteries from age-paired diabetic (C) and non-diabetic (D) patients. Data are mean + SEM.


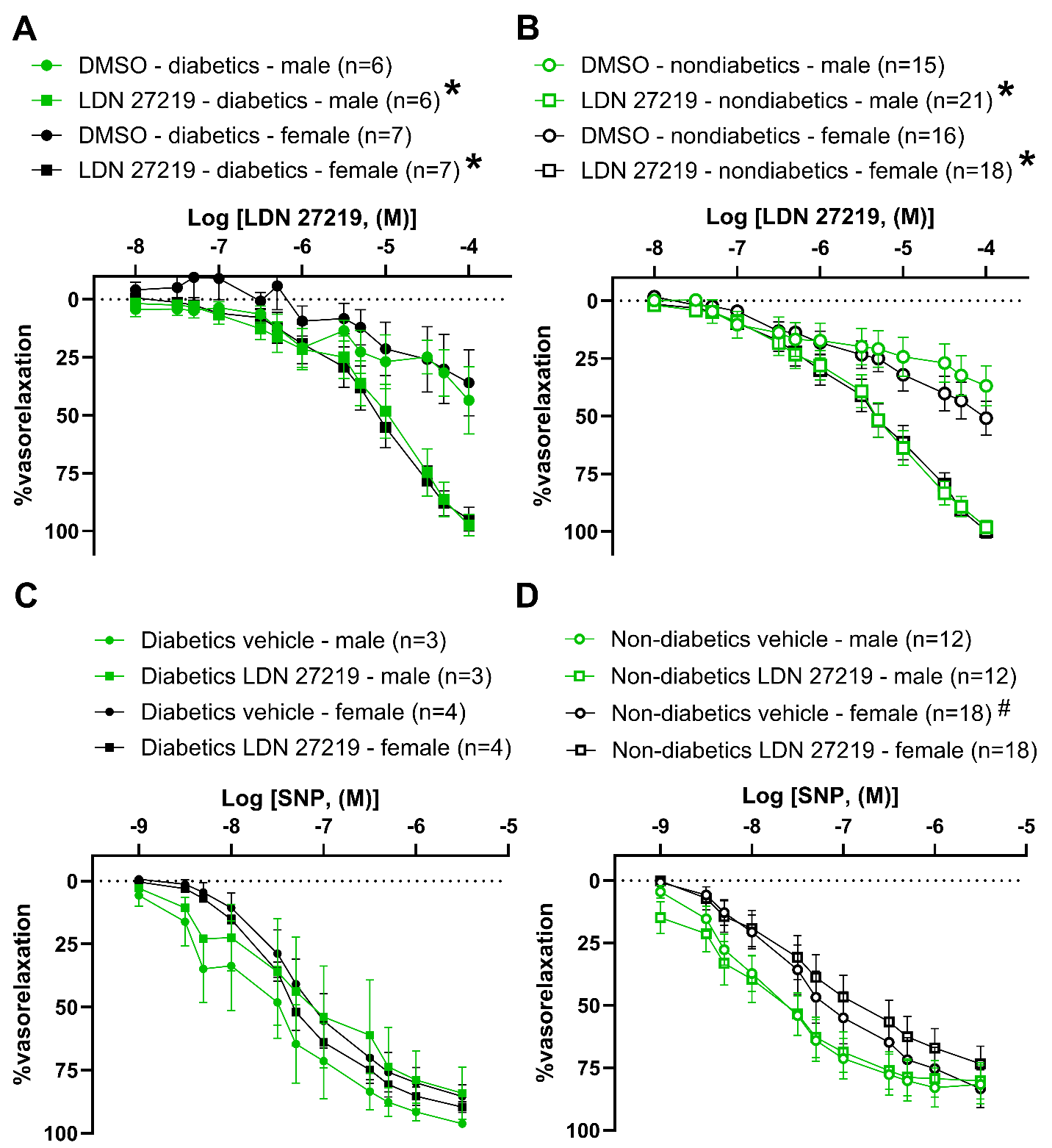


**Supplemental Figure S3**. **A-B.** Average concentration-response curves for LDN 27219 or vehicle (DMSO) in U46619-contracted arteries from type 2 diabetic (A) and all non-diabetic patients (B) divided by sex. The protocol corresponds to the one portrayed in Figure 2 in the main manuscript. **C-D.** Average vasorelaxations to SNP in Phe-contracted arteries from type 2 diabetic (C) and all non-diabetic (D) patients incubated with either vehicle or LDN 27219 (3x10^-6^ M). Data are mean + SEM. Differences were evaluted by two-way ANOVA. *p < 0.05 vs. DMSO control. # p<0.05 vs. Non-diabetics vehicle – male. Data are average ± SEM.

**
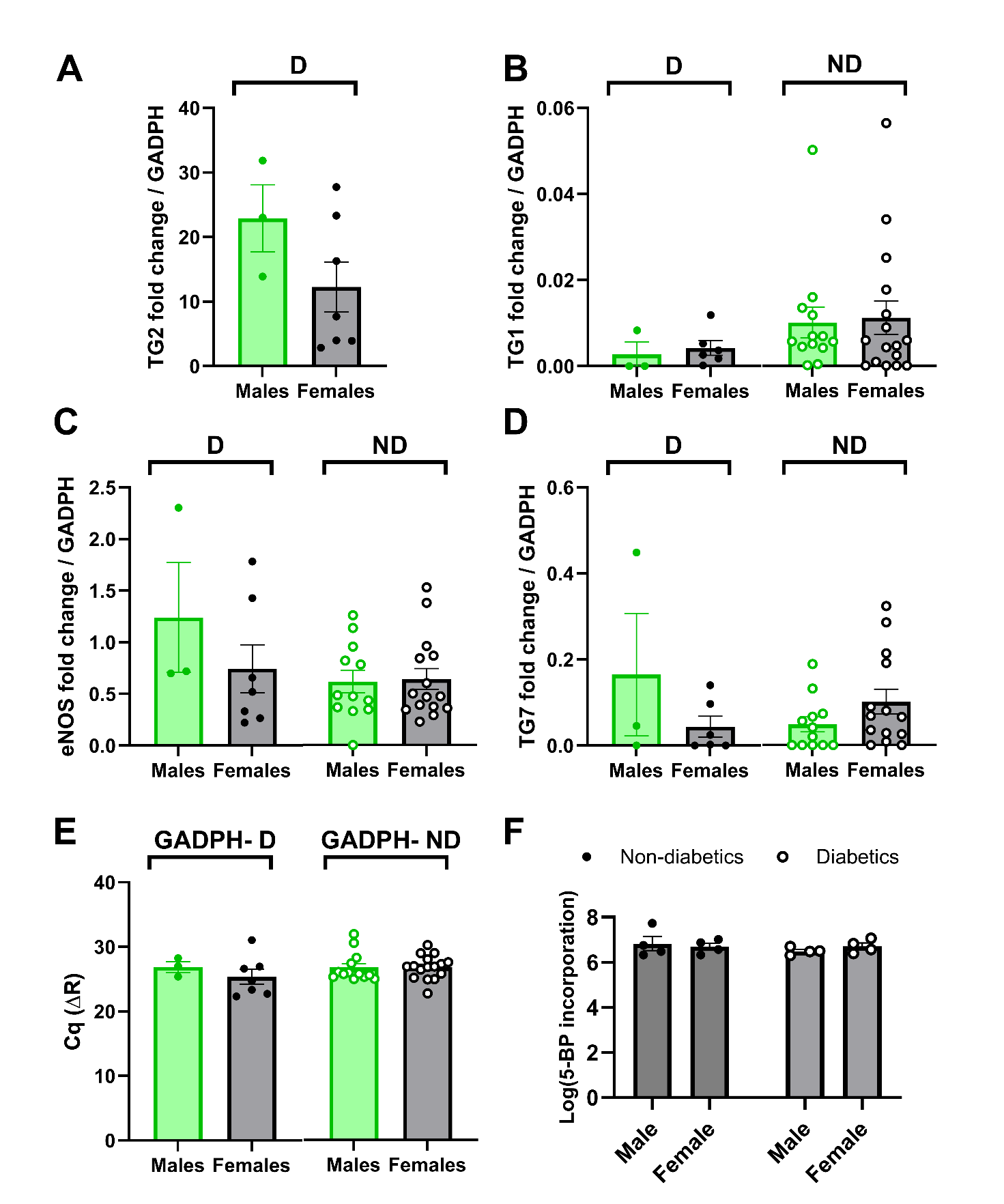
**

**Supplemental Figure S4**. Quantification of mRNA expression by RT-qPCR of TG2 (A), TG1 (B), eNOS (C), TG7 (D) and GADPH (E) in subcutaneous arteries of all studied patients divided by sex. **F.** Transamidase activity quantified by 5-BP incorporation, average log(5-BP incorporation) (arbitrary units) in subcutaneous artery samples of diabetic and non-diabetic patients divided by sex. D: Diabetics. ND: Non-diabetics. Data are averages ± SEM.
